# Characterization of the Cassava Mycobiome in Symptomatic Leaf Tissues Displaying Cassava Superelongation Disease

**DOI:** 10.3390/jof9121130

**Published:** 2023-11-23

**Authors:** Angela Alleyne, Shanice Mason, Yvonne Vallès

**Affiliations:** Faculty of Science and Technology, The University of the West Indies, Cave Hill Campus, Bridgetown BB11000, Barbadoskyvovas@gmail.com (Y.V.)

**Keywords:** microbiome, cassava, *Elsinoë*, *Sphaceloma*, superelongation disease

## Abstract

Superelongation disease (SED) is a fungal disease that affects cassava in the Caribbean. The symptoms include the appearance of dry necrotic spots and lesions on the leaves, which may severely affect the plant yield. However, the primary causal pathogen is difficult to culture and isolate in the lab because of its slow growth and potential contamination from faster-growing organisms. In addition, the leaf symptoms can be confused with those caused by other pathogens that produce similar necrotic spots and scab-like lesions. There is also little or no information on the contribution of endophytes, if any, to disease symptoms in cassava, a plant where the disease is prevalent. Therefore, this study aimed to characterize the fungal communities in cassava associated with SED symptoms by analyzing gross fungal morphology and performing metagenomics profiling. First, several individual pathogenic fungi were isolated and cultured from diseased cassava leaf tissues from seven locations in Barbados (BB). Both culture isolation and molecular community analyses showed the presence of several other fungi in the disease microenvironment of symptomatic cassava leaves. These included *Fusarium*, *Colletotrichum*, and *Alternaria* species and the suspected species *Elsinoë brasiliensis* synonym *Sphaceloma manihoticola*. Additionally, a community analysis using ITS2 amplicon sequencing of 21 symptomatic leaf tissues from BB, St. Vincent and the Grenadines (SVG), Trinidad and Tobago (TT), and Jamaica (JA) revealed that the disease symptoms of superelongation may also result from the interactions of fungal communities in the mycobiome, including *Elsinoë species* and other fungi such as *Colletotrichum*, *Cercospora*, *Alternaria*, and *Fusarium*. Therefore, we suggest that examining the pathobiome concept in SED in the future is necessary.

## 1. Introduction

Cassava (*Manihot esculenta* Crantz) is native to Latin America and the Caribbean [1], and its edible tubers are a staple food for approximately 800 million people worldwide [1,2]. In some Caribbean islands, namely BB, TT, and JA, cassava is planted as a single crop (pure stand) or intercropped with vegetables, legumes, or other plants. Intercropping is usually practiced by small-holder farmers who do not produce commercially and is more prevalent in SVG [3]. Cassava is an essential component of food security in these islands [2,4], but it is susceptible to many fungal diseases [5].

The vegetative propagating material of cassava can be severely affected by pathogenic fungi, specifically the foliage, stems, and root tubers. Important fungal pathogens, including *Cladosporium* spp., *Colletotrichum* spp., *Magnaporthe oryzae*, *Fusarium* spp., *Cercosporidium henningsii*, and *Botrytis cinerea* [6], appear frequently in cassava cultivations. In susceptible cassava varieties, cassava anthracnose disease (CAD) caused by *Colletotrichum gloeosporioides* presents as stem and petiole cankers, followed by extreme necrotic lesions and defoliation, resulting in crop losses of more than 30% [7,8]. Similarly, *C. henningsii*, synonym *Mycosphaerella henningsii*, which causes cassava brown leaf spot disease, is reported to have caused a yield decline of 17–30% in Africa, South America, and Asia [9,10,11]. Another cassava fungal disease of economic interest in Latin America and the Caribbean is the SED, caused by the fungal pathogen *E. brasiliensis* [5,12]. In South and Central America, there are reports of yield losses attributed to SED of 80% in vulnerable cultivars [12,13] and reports of its presence in BB and TT [14,15] but not elsewhere in the island chain.

**Figure 1:**
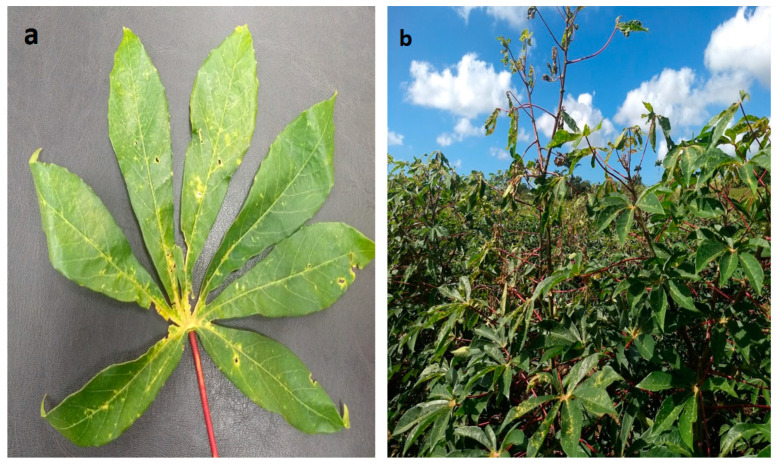
Cassava SED symptoms. In (**a**), irregular chlorotic spots, leaf curling, and shot holes are visible on the lamina. In (**b**), evidence of internode elongation is seen in the field with shoot-tip dieback.

The characteristic symptoms observed in SED are due to the *Elsinoë* or *Sphaceloma* de Bary species. These are usually tiny, light-colored, chlorotic circular leaf spots on the leaf surface or are sometimes irregularly shaped [5,13]. Some leaf spots have a necrotic center region that eventually will form an open area or “shot-hole” when dry (similar to shot-holes caused by cassava brown leaf spot). As the disease severity increases, cankers or scabs become visible on petioles, stems, and leaf veins. The internodes elongate and extend above the canopy [12,16]. Internode elongation usually occurs after dieback, in which the shoots die from the tip inwards [13] (Figure 1).

Morphological and laboratory culture methods are used to identify fungal plant pathogens. These include observing signs in symptomatic plants, such as fungal spores or fruiting bodies, and a detailed microscopic examination of their sexual structures [17,18]. However, these approaches have several limitations, including the low recovery rates of some organisms [19]. For unculturable pathogens, molecular diagnostics or biosensor analyses are employed for characterization [6,18].

Several molecular markers such as RNA-polymerase II (RPB2), translation elongation factor (TEF), and β-tubulin II (TUB) are used to identify unknown fungi at the species or genus level [20,21]. However, in mycobiome-wide association studies (MWAS), the internal transcribed spacer gene (*ITS*) of ribosomal DNA (rDNA) is a frequently targeted barcode gene because it often allows accurate genus and species identification [6,22,23]. Although rare species and taxonomically incomplete databases may cause an underestimation of microbial diversity, this method remains a gold standard in metagenomic studies [24,25,26].

The community structure of the mycobiome in SED in cassava is unknown. Further, the fungal communities in the disease microenvironment are also unknown, and no previous studies have compared SED in the Caribbean islands affected by the disease. The mycobiome represents all the fungi or fungal communities in a particular ecological plant niche in the plant metagenome [26]; however, the interactions or functions of fungal endophytes in the plant phyllosphere, especially in symptomatic plant tissues, have not been sufficiently documented [6,27]. The mycobiome is critical to maintaining a healthy plant and can guide disease management in plants such as cassava [26]. This study aimed to identify fungal endophytes and phytopathogens associated with symptomatic disease in cassava in the Caribbean islands using culture-dependent methods and metagenomics, explicitly focusing on the mycobiome. We adopted a community-wide approach to characterize further species richness and taxonomic correlations of fungi with disease symptoms in cassava fields on the Caribbean islands. Our goal was to explain whether SED was present in most cassava-producing areas in the Caribbean islands and to describe the organisms associated with the disease by examining the mycobiome of cassava leaves in four Caribbean islands. Understanding which fungal communities are present in symptomatic tissues may improve the management of SED in cassava.

## 2. Materials and Methods

### 2.1. Study Area and Field Collection of Diseased Leaves

We collected symptomatic leaf samples between December 2018 and January 2019 from six-month-old cassava plants in the field. As previously described, we collected leaves with small chlorotic spots from plants displaying typical SED symptoms, including shot holes and internode elongation (if visible) [16]. The leaf samples were placed in a clean zip-lock bag, stored in an ice cooler, and transported to the lab for fungal isolation and DNA extraction within 24 h. Fungal isolations, pathogenicity tests, and DNA extractions from symptomatic leaf tissues were conducted in BB, where SED was previously reported [28]. In contrast, in TT, JA, and SVG, only DNA was extracted directly from symptomatic cassava leaves because of phytosanitary constraints on the movement of diseased plant materials (Table 1). The average plot size varied from 557 m^2^ in SVG to 80 ha in JA, with TT having an average plot size of 1 ha and BB 2 ha. In each of the twenty-one fields we visited, we collected forty-five leaves from three randomly selected plants located in three different sectors representing a cross-section of the field, and we selected five leaves per plant. The selected leaves from each plant were pooled and used for DNA extraction. Finally, the twenty-one pooled leaf samples used for DNA extractions represented a field in each island sampled: JA, SVG, BB, and TT (Table 1). Asymptomatic first leaves taken from a cassava stake dipped in fungicide were used as a negative control for cultural identification of fungi.

### 2.2. Isolation of Fungi from Symptomatic Leaf Tissues

We collected leaf samples from seven field locations in BB from plants exhibiting SED symptoms for the cultural isolation of fungi (Table 1). We used a sterile scalpel blade to cut three symptomatic leaves from cassava into 2 cm squares. To suppress epiphytic microbes, we briefly surface disinfested each square of plant tissue through consecutive washes for 1 min with 70% ethanol and 1% bleach solution [16]. After surface sterilization, the tissues were rinsed quickly with sterile distilled water and placed on sterile absorbent paper to blot dry. They were then placed on potato dextrose agar (PDA-Sigma Aldrich, St. Louis, MO, USA) amended with 25% lactic acid (Sigma Aldrich, St. Louis, MO, USA) and streptomycin sulphate (Sigma Aldrich, St. Louis, MO, USA) at 100 ppm [16] for the culture of the suspected phytopathogen. A single colony of each suspected phytopathogen from the leaf tissue was subcultured on PDA and grown under dark conditions for one to two weeks at 30 °C. The cultures were stored longer in a 20% glycerol (Sigma Aldrich, St. Louis, MO, USA) solution in the freezer at −20 °C. We collected fifty-three single fungal isolates in BB and stored them long-term. 

We carefully observed the general fungal morphologies after culture by examining their colors, textures, and shapes [5,16]. The gross morphological characteristics of the suspected phytopathogens believed to be *E. brasiliensis* were then compared with the features of the available reference strain 395825, Centre for Agricultural and Biosciences International (CABI) [5]. Each isolate was subcultured onto PDA and described based on cultural morphologies.

### 2.3. Pathogenicity Tests of Suspected Isolates

We inoculated 20 plants with the isolate suspected to be *E. brasiliensis* (see Section 2.2) in the shade house at the University of the West Indies, Cave Hill Campus, BB. Four mature leaves were inoculated with the suspected *E. brasiliensis* isolate. For each plant, we surface sterilized the selected leaves with 70% ethanol, and then rinsed them with sterile distilled water and swabbed them with a pure isolate culture using sterile swabs. Similarly, two control leaves were surface sterilized and inoculated using sterile distilled water. Immediately after inoculation, we simulated a humidity chamber by covering each plant with a clear plastic bag fastened at the base. We removed the humidity chamber 24 h later, watered the plants every other day, and observed them daily for disease symptoms for 21 days. We replicated the experiment and recorded disease symptoms using a modified scale (Table 2) based on a previously reported SED disease scale [13].

### 2.4. DNA Extraction from the Leaf Tissues

We extracted DNA from the leaf tissues obtained from 21 field locations in BB (7), SVG (4), TT (3), and JA (7). We used the method described by the manufacturer for DNA extraction with the Wizard^®^ Genomic DNA Purification Kit (Promega Corp. Madison, WI, USA) to extract DNA from the leaves collected by field sampling. We then pooled the leaf tissues derived from 45 leaves [5]. Each sample was rehydrated in 100 µL of DNA rehydration solution and incubated for 1 h at 65 °C. Extracted DNA was then quantified using Promega Quantus™ Fluorometer (Promega Corp., Madison, WI, USA) for PCR amplification and genomic sequencing.

### 2.5. Amplicon Sequencing and Mycobiome Analysis

We conducted metagenomic studies on the symptomatic leaf tissues of TT, JA, and SVG, for which fungal culture was impossible. However, we performed both culture and mycobiome studies on the samples from BB (Table 1).

The following PCR primers ITS1-F ‘CTTGGTCATTTAGAGAGAGAGTAA’ and ITS2-R ‘GCTGCGTTCTTCATCGATGC’ (Integrated DNA Technologies, Inc., Coralville, IA, USA) were used to amplify the entire ITS region. For the PCR, we used genomic DNA (5 ng), AmpliTaq Gold^®^ DNA Polymerase (5 U/µL), PCR Buffer (10×-AmpliTaq^®^ Gold), MgCl_2_ (25 mmol/L) (Thermo Fisher Scientific, Waltham, MA, USA), PCR primers (20 µM), and dNTPs (10×) in a final volume of 25 µL. The reaction conditions were initial denaturation, 95 °C for 1 min; 35 cycles of denaturation at 94 °C for 1 min; annealing, 60 °C for 2 min and extension at 72 °C for 2 min; and final extension stage at 72 °C for 10 min [5,29].

After PCR amplification, the amplified products were purified with a Qiagen Qiaquick PCR purification kit (Qiagen LLC, Germantown, MD, USA), followed by library preparation. Library preparation and Illumina MiSeq sequencing were conducted using a NEBNext^®^ Ultra II kit for Illumina (New England Biolabs, Ipswich, MA, USA) at GENEWIZ, Inc. (GENEWIZ Inc., South Plainfield, NJ, USA). Afterwards, the library fragments were amplified with specific ITS2 forward (F) and reverse (R) primers, namely ‘GTGAATCATCGARTC’ (F) and ‘TCCTCCGCTTATTGA’ (R). Besides the ITS2 target-specific sequences, the primers also contained adaptor sequences, enabling the selection of the library in preparation for downstream NGS sequencing on the Illumina MiSeq platform (GeneWiz ITSEazy protocol; GENEWIZ Inc., South Plainfield, NJ, USA). Approximately 50–100 ng of genomic DNA was amplified and sheared. End repair, phosphorylation, and dA-tailing were performed in a combined reaction for each sample for 30 min at 20 °C, followed by thermal enzyme inactivation at 65 °C for 30 min. Adaptor ligation was conducted using the NEBNext^®^ ligation master mix, the NEBNext ligation enhancer, and NEBNext dual-indexed IDT adaptors (New England Biolabs, Ipswich, MA, USA). The ligation reactions were incubated for 15 min at 20 °C. Ligated libraries were purified using two rounds of magnetic SPRI bead purification (0.7× volume). Next, library validation was conducted with an Agilent 2100 Bioanalyzer (Agilent Technologies, Palo Alto, CA, USA) and quantified with a Qubit 2.0 Fluorometer (Invitrogen, Carlsbad, CA, USA). The library was sequenced by paired-endreads with a 2 × 300/250 configuration, followed by image analysis and base calling with the MiSeq Control Software v.3.0 (GENEWIZ Inc., South Plainfield, NJ, USA).

### 2.6. Data Analysis

We used the Quantitative Insights into Microbial Ecology (QIIME 2) data analysis package [30] for library analysis. Chimeric sequences were filtered out and removed with the UCHIME algorithm. The remaining sequences were clustered by VSEARCH (1.9.6) into operational taxonomic units (OTUs) by searching against the UNITE ITS database (http://qiime.org/home_static/dataFiles.html, accessed on 8 August 2019) at 97% sequence identity. A single representative sequence for each OTU was used to annotate at the taxonomic level using the RDP classifier at a confidence threshold of 0.8. We conducted additional sequence analysis in BLAST to verify the taxonomy, whereas unclassified sequence data were omitted from further analysis. Samples were rarefied when calculating alpha diversity indices (richness and Shannon) in R using the vegan package, accessed on 1 August 2023. Beta diversity was visualized by Canonical Correspondence Analysis (CCA), and we used an ANOVA test to determine statistical significance. Statistical analyses were carried out in RStudio v2.15. 3 [31].

We conducted taxonomic composition levels (phylum, class, order, families, genus, species) by cluster analysis with the Ward algorithm on a Bray–Curtis dissimilarity matrix to quantify abundance dissimilarities between the islands, and we visualized this on a heatmap. We used ANOVA and a differential analysis based on the negative binomial distribution (DESeq2) to test for the statistical significance of differential abundances. All data were deposited in ENA as PRJEB46406.

## 3. Results

### 3.1. Description of Pathogenic and Non-Pathogenic Fungi in Symptomatic Cassava Leaves

**Figure 2:**
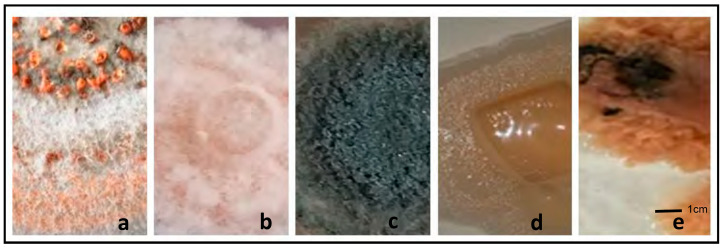
Various fungi were isolated from cassava leaves grown on PDA after one week. Some isolates resembled *Colletotrichum* spp. (**a**), *Fusarium* (**b**), *Alternaria* (**c**), and *E. brasiliensis* (**d**). The reference strain 395825 (**e**) was used for comparison.

Various fungal morphologies were observed when isolating *E. brasiliensis* from symptomatic cassava leaves. Our observed fungal morphologies included many isolates with aerial mycelium with a woolly like texture, but some were mucoidal. Some of the primary recurring colony morphologies seen on PDA were as described in the literature and resembled those of *Colletotrichum*, *Fusarium*, and *Alternaria*, respectively (Figure 2a–c).

For example, we inferred from observing white cottony mycelia with pink pigmentation that *Fusarium* species were present in the infected tissues. Those cultures that we identified from their morphologies as *Colletotrichum* species had a mixture of off-white and pink colors with thick mycelia and dark-grey, or white-gray, woolly, and white mycelia with a salmon color and oozing conidia in the center of the culture. Some olive-green colonies resembled *Alternaria* because of their felted to woolly appearance and wavy margins on PDA (Figure 2).

*Elsinoë brasiliensis* was also suspected based on the morphological observations in symptomatic leaves and SED-like disease symptoms on stems and petioles. However, our suspected morphotype (Figure 2d) differed significantly from the reference *E. brasiliensis* (CABI 395825) in several features at the macroscopic level (Figure 2e). The isolates from BB were smooth, mucoid, had pale-orange colors and had an irregular shape (Figure 2d). Interestingly, although the reference strain CABI isolate 395825 of *E. brasiliensis* was rough-shaped and pale orange, it also had black regions and displayed a raised, gummy texture with deep fissures, which were not observed in the BB isolates. The *E. brasiliensis* CABI reference isolate 395825 also had septate hyphae with a few small, ellipsoidal, and hyaline conidia. No culturable fungi were observed on PDA in the control leaf sample.

On infection with the suspected *E. brasiliensis* isolate, minute flecks appeared all over the leaf surface and were scored at disease severity 1.0. As the disease progressed and by the end of day 21, all the infected plants with minute flecks became more defined with chlorotic spots with a severity index of 1.5 (Table 2). However, some plants developed these symptoms earlier than others.

### 3.2. Characteristics of the Cassava Leaf Mycobiome from Symptomatic Tissue

After quality filtering, 447,244 reads were retained, with an average number of reads per sample of 21,297 and maximum and minimum values of 126,077 and 1238, respectively. These reads were clustered into 499 OTUs based on 97% similarity. Further filtering by removal of unclassified reads and OTUs with less than two counts resulted in a final dataset comprising two phyla, 14 classes, 26 Orders, 42 families, 56 different genera, and 51 species.

The phylum Ascomycota showed the greatest dominance (relative abundance (RA) = 79–100%) across the sample sites. Contrastingly, the RA for Basidiomycota was low (5.4%). Mucoromycota (Genus: *Poitrasia*) was only observed in one sample site in SVG, with an RA of 0.005%, and was therefore omitted from further analysis (Supplemental Figure S1).

Among the Ascomycota, Dothideomycetes was the predominant class (mean abundance = 65.46%) and represented almost a quarter of all fungi present (23%); most Dothideomycetes belonged to two families, Pleosporaceae (17%) and Mycosphaerellaceae (15%) (supplemental data Figure S2). However, their distribution was different. Mycosphaerellaceae were primarily found in SVG and TT, whereas Pleosporaceae were predominant in JA and BB, at similar levels (supplemental data Figure S2). Debaryomycetaceae was the main family in SVG, whilst Elsinöaceae predominated in symptomatic cassava leaves in TT.

**Figure 3:**
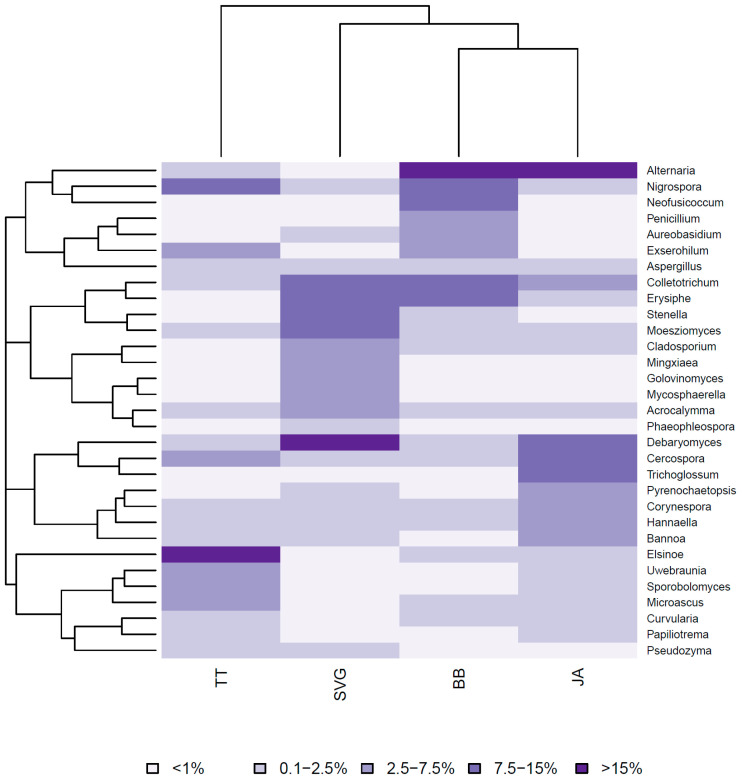
Genera composition in BB, JA TT and SVG from diseased cassava leaves. Only relative abundances greater than 1% are shown.

When we compared the mycobiome composition of symptomatic cassava leaves from each island at the genus level, predominantly high levels of *Elsinoë* were observed in the leaves from TT (>15%), but in those from BB and JA, much lower quantities were observed (2.5–7.5%). *Elsinoë* spp. Was not observed in the leaves from SVG (Figure 3). The presence of large quantities of *Debaromyces* in SVG sites contrasted with the absence of *Elsinoë* there. At the same time, the large amounts of *Elsinoë* in TT contrasted with the low proportions of *Debaromyces* (in TT and BB) (Figure 3).

**Figure 4:**
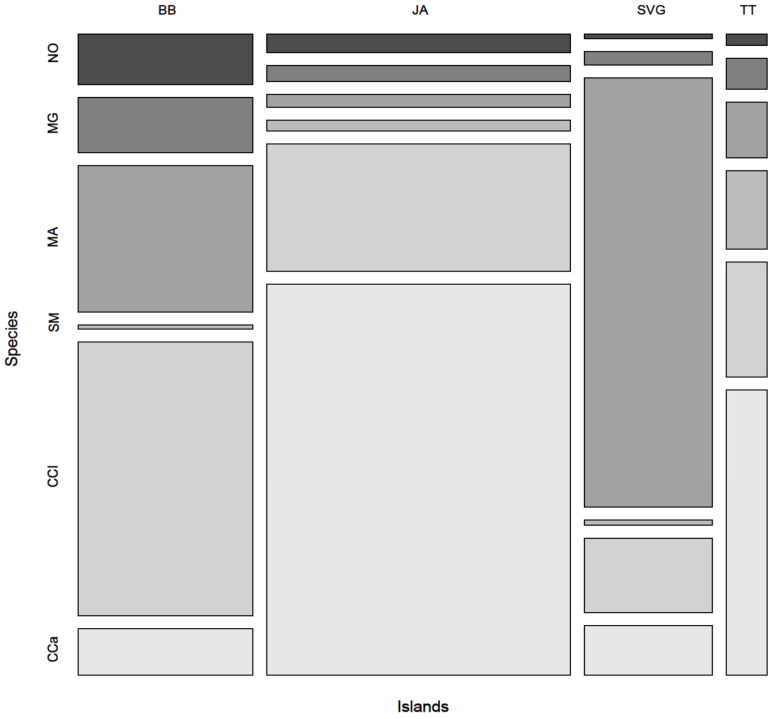
A mosaic plot showing the distribution of the only six species observed in BB, JA, SVG and TT. A separate column represents each island, and the width of the columns indicates the relative abundance or proportion of those six species for each island. The height of the box shows the relative abundance of the species compared to the other five. The color of the boxes corresponds to the different species as they appear in the first column in BB. NO: *N. oryzae*, MG: *M. gramineum*, MA: *M. aphidis*; SM: *S. marina*, CCl: *C. cliviae*, CCa: *C. canescens*.

*Cercospora canescens* was present at the species level in all four islands and, on average, was the most abundant species (9.15%). Yet, it was absent in specific sites in some areas in BB and SVG, where occurrences were generally low (Figure 4). Also, *C. cliviae* was present in all four island locations, occurring more frequently in JA and BB (7.5–15%) (Figure 4). Six species were present on symptomatic cassava on all four islands, namely *Nigrospora oryzae*, *Myrothecium gramineum*, *Moeziomyces aphidis*, *Symmetrospora marina*, *C. cliviae*, and *C. canescens* (Figure 4). Amongst the species not observed in all four islands was *E. corylii*, which was not detected in SVG but was revealed to be the dominant species in the mycobiome at four sites spanning three islands, namely, JA (JAWa2), TT (TTPe and TTF3), and BB (BBH1). TT had the highest prevalence (~54% on average). Other important species included *Curvularia lunata*, found in 11 of the 21 sites, especially in JA; *Hannaella senensis*, detected in 10 of the 21 sample sites, both of which were absent in SVG; and *Cladosporium sphaerospermum*, observed in 7 sites, which was most abundant in SVG and BB, low in JA, and absent from TT.

The Shannon–Weaver diversity index varied in each island, with SVG having the largest range differences (0.69–2.38) and BB the lowest (0.96–1.77) (Table 3). Nonetheless, statistical analysis of the data resulted in no significant difference among the islands (ANOVA: *p* = 0.702; F_3,17_ = 0.477) in species evenness. Likewise, although differences in species richness were evident among the islands, it was not statistically significant (ANOVA: *p* = 0.630; F_3,17_ = 0.589).

**Figure 5:**
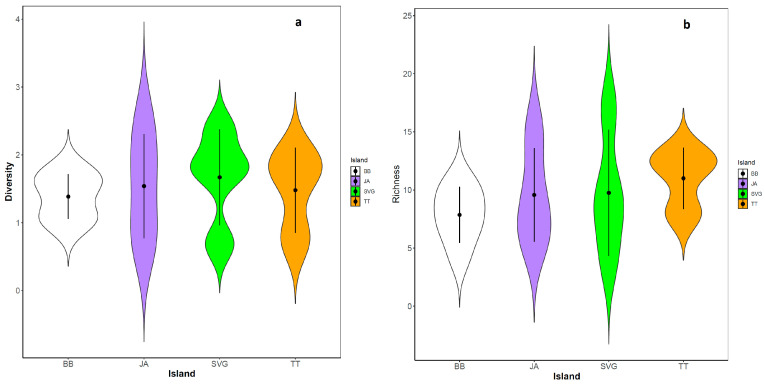
Violin plots showing fungal diversity (**a**) and fungal richness (**b**) in cassava plants from different Caribbean islands, BB, JA, SVG and TT.

**Figure 6:**
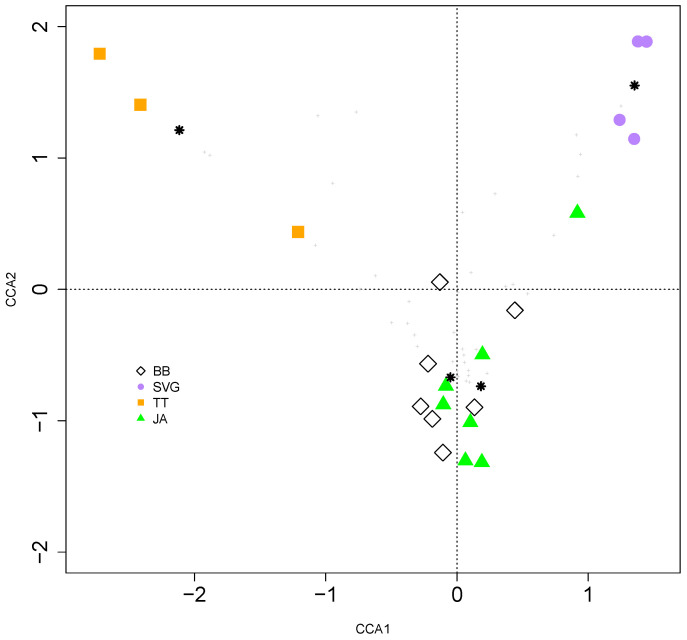
Canonical Correspondence Analysis (CCA) of fungal communities in cassava plants from different Caribbean islands (BB; JA; SVG; TT). While the main axis (CCA1) separates samples from TT and SVG, the second axis (CCA2) separates the latter samples from those of BB and JA. * cluster groups for each island.

Indeed, the violin plots for species counts in each community indicated that fungal diversity (species abundance and evenness) was broad everywhere except in TT and SVG, as shown by the more uneven distribution of fungal diversity in the bimodal plot (Figure 5). While diversity and richness indices showed a wide distribution of fungi infecting cassava within each island, CCA showed significant geolocational differences between the islands (*p* = 0.001) (Figure 6). As shown in Figure 6, the main axis (CCA1) entirely separates the TT and SVG samples, whilst the CCA2 axis underlines a closer relationship between the isolates from BB and JA compared with the ones from TT and SVG, which appear separate and distinct from each other (Figure 6).

Of note, two groups of fungi were of significant concern when examining positive fold changes in the differential abundances of the fungal communities between the islands. These were *Neofusicoccum* species in BB and *Alternaria* species in all locations. Significant abundance differences (*p* < 0.05) were evident for these two fungal groups (Table 4).

## 4. Discussion

Cassava is known to harbor fungal genera belonging to many species as endophytes and/or plant pathogens [32,33,34,35]. Our study confirmed this as we isolated pathogenic fungi from *Alternaria*, *Elsinoë*, *Neofusicoccum*, *Fusarium*, *Mycosphaerella*, and *Colletotrichum* genera, among others. However, there are very few studies on the fungal communities in cassava [23,32], especially on the cassava leaf mycobiome and the mycobiome in infected, symptomatic tissues. Using traditional means, we observed various fungal morphologies from SED-like symptomatic cassava leaves (Figure 2). These included fungi that looked like *Colletotrichum* and *Fusarium* species, two well-known plant pathogens that can also cause diseases in cassava [35,36], and endophytes such as *Alternaria* [23,32], a leaf spot-causing parasitic fungus that affects plants that may also have a toxigenic effect in humans [37]. According to the emerging pathobiome concept of plant disease, based on the observation that symbiotic microbial communities, including pathogenic microorganisms, may exist in plants, several organisms in symptomatic tissues rather than only a single one may contribute to the disease state at different times in plants [38,39].

We found that of the OTUs examined, Dothideomycetes was the predominant fungal class (65%) among the ascomycetes. Similar results are typical of the mycobiome in crop plants [26,27,40] as Dothideomycetes is the primary fungal class comprising many important pathogens in the fungal kingdom [41]. We also showed that among the Dothideomycetes, Pleosporaceae, Mycosphaerellaceae, and Elsinöaceae were the predominant families. In TT, Elsinöaceae was most abundant and was particularly interesting as it is the designated single causal agent of SED [16,28,35] and includes *E. brasiliensis*. In addition, at each location, we observed SED-like symptoms in the plants. However, despite similar morphological symptomatic characteristics, the abundance of SED was not consistent across sites and islands. Morphologically, shot-hole symptoms in cassava brown leaf spots are similar to those in SED (Figure 1) and may account for the variation seen when using morphology alone as a determining factor of SED.

Hence, there is a need for more robust methods of identification, such as molecular diagnostics. In JA, the soil saprotroph *Alternaria* occurred in high abundance, as seen from the molecular studies, and although present in BB, it was less common in both the fungal cultures and molecular analyses. Mycosphaerellaceae are largely plant pathogens that are reported as the causal agents in bean and cassava leaf spot diseases and Sigatoka in bananas [21,35,42]. Community class organization suggested that Pleosporaceae were mainly present in BB and JA, whereas Mycosphaerellaceae were observed in TT and SVG (Figure 3). This distinction may be one of several factors that accounted for the community grouping by CCA, which clustered JA and BB fungal communities together and separated both TT and SVG fungal communities in cassava. However, of the six species we observed on all four islands, we saw distinctly different relative abundance and distribution patterns in the mosaic plot in Figure 4. So, species abundance alone could not account for the cluster patterns shown in Figure 6 by CCA. Several other factors may account for the community grouping by CCA, which clustered JA and BB fungal communities together in contrast to TT and SVG. The former islands are geographically separated into the Greater and Lesser Antilles in the Northern and Southern Caribbean, respectively. Therefore, the proximity of BB to SVG and TT makes differences in environmental factors such as long-distance spore dispersal and the use and transport of similar planting materials within the island chain unlikely explanations. Moreover, the difference in cropping practices between SVG and the other islands TT, BB, and JA, where more commercial stands are prevalent, also does not explain these differences given the geographical proximity of TT, BB, and SVG in the Southern Caribbean. Still, higher disease burdens in neighboring commercial cropping systems on each island may influence the cluster patterns seen here since JA and BB have distinct cassava agro-industries. However, this is unknown. Therefore, the agroecology of fungal pathogenesis in cassava in the Caribbean islands must be explored further.

The cassava leaf mycobiome across all four islands contained 51 identified fungal species, of which only 11.75% were found in all four islands, though, interestingly, not in all sites. In addition, some of these species were clearly at different abundances, in some cases comprising up to 45% of the species relative abundance, as observed for *C. canescens* in JA, while in TT, they only comprised 6% of the total species abundance (Figure 4). Microbial diversity may be influenced by differences in environment, soil conditions, and farm practices [43,44]. Some studies show that microbial variation in the phylloplane is directly related to acute changes in abiotic environmental factors [44,45,46,47]. Others have shown that the host genotype and its development stage are also critical biotic factors in the selection of microbes in the mycobiome [48]. Abiotic factors, including rain and wind flow, help to spread fungal conidia and contribute to interval variations seen in the RA of resident fungi on the leaf. All these factors may explain the similarities and differences observed in the cassava mycobiome on each island, with each site having slight differences in microclimate and, similarly, differences in cassava cultivars grown. We hypothesize that many species unique to the mycobiome in SVG may be due to differences in cultural practices adopted by the mixed-type small holdings for the cultivation of a variety of local cassava landraces there in comparison with the agricultural practices common to TT, BB, and JA, where mono-crop cultivation is prevalent because the cassava industries there are more significant and more commercial [49]. Farmers in SVG plant other crops, including pumpkin, sorrel, and yam, alongside cassava on smaller land holdings. This type of inter-cropping system may cause introgression, infection, and even adaptation by various other microbes into nearby cassava plots. The variety of fungi seen in these four islands provides some evidence for adopting different agronomic management strategies for cassava in the island chain based on the traditional and common practices of farmers, their commercial interests, and the dominant resident microbes or pathogenic agents that threaten the crop in specific island locations.

It is also possible that some species in low abundance in this study may represent a pioneer or rare species. Unfortunately, no published studies have identified associated mycoflora in the microenvironment of cassava leaves, especially in the Caribbean islands. *C. gloeosporioides*, *C. canescens*, *N. oryzae*, *M. aphidis*, *M. gramineum,* and *Symmetrospora* spp. were present in the four islands we studied. These may represent elements of a representative community of pathogenic and non-pathogenic organisms that function together, affecting the disease dynamic of SED or other known fungal diseases in cassava in the sampled Caribbean islands. Of the species identified, *C. gloeosporioides* and *Cercospora* spp. are known pathogens in cassava; however, some other species are not known to form pathogenic associations in cassava and may simply be resident endophytes. Some of these can also cross the human–plant interface, such as species belonging to the genera *Aureobasidium* and *Exerohilium* [50,51].

There are previous reports in 2007 and 2008 in TT and BB, respectively, of these disease symptoms in cassava [15,28]. The damage caused by the pathogen is highly variable. Some plants exhibit lengthening of their internodes, especially during the rainy season, whereas others do not [14,15]. Although the SED symptoms observed were characteristic of fungal pathogens belonging to *Elsinoë* species, the primary symptoms can sometimes be associated with other fungal diseases of cassava [35,36]. In addition, field symptoms of internode elongation and secondary symptoms of SED caused by the overproduction of gibberellic acid produced by *Elsinoë* species are not always observed in mature cassava plants [15,28,52]. In this study, although the main features of the isolates from the symptomatic tissues corresponded to some morphological descriptions of *E. brasiliensis* from earlier studies, such as the presence of the pale-orange colonies with mucoidal textures and black-pigmented areas when aged, they differed from those of the reference strain used (CABI 395825) for *E. brasiliensis* (Figure 2). Interestingly, single gene sequencing identified this fungal isolate from BB as an *Aureobasidium pullans* NCBI accession KT352844 (A. Alleyne pers commun.), a yeast-like fungal species that is ubiquitous in the environment and is commonly found as a minor saprophytic fungal endophyte on leaves [51,53]. Although symptomatic cassava leaves displayed symptoms of SED in this study, a complex community of other fungi, including fungal endophytes or other *Elsinoë* species, may have contributed to these symptoms. Moreover, Legg and Alvarez [4] reported that multiple species of *Elsinoë* may affect cassava; therefore, other species of *Elsinoë*, such as *E. corylii*, which we detected in the cassava mycobiome, could contribute to the SED symptoms observed in TT and BB.

Cassava has a complex heteroploid genome; many local landraces and commercial cultivars grown throughout the Caribbean are dominated by Brazilian genotypes [54]. The plant genotype may condition the mycobiome composition within plant species [48] in the Caribbean islands because various cassava cultivars are usually planted across the island chain. Some, such as Bra 383, MMex, and CIAT, are genetically improved accessions against SED. The small genetic changes represented by these specific cultivars could also account for variation in the cassava mycobiome among the islands. As the pathobionts concept in plant pathology evolves and disease diagnosis becomes less reductionist, a broader sampling of several tissues or plant organs from cassava, including both symptomatic and asymptomatic tissues from the tubers and stems over the plant growth cycle and in different environmental conditions, may be necessary to conclusively determine the main contributors or the network alliances in the microbiota associated with SED in symptomatic cassava in the Caribbean islands.

## 5. Conclusions

While symptoms of superelongation disease persist in cassava, *Elsinoë* species seem to form only one of many microbes contributing to the disease dynamic in the mycobiome in diseased tissues, accounting for only 1% in some instances. Among the islands, *Elsinoë* was absent in St. Vincent and the Grenadines, occurring most frequently in Trinidad and Tobago and less so in Barbados and Jamaica. Significantly, six species mainly contributed to those observed in all the islands, including pathogens such as *C. canescens* and *C. cliviae* and endophytic yeasts including *M. aphidis*, especially in St. Vincent and the Grenadines and Barbados. However, these alone do not account for pathogenic differences in cassava in the island chain. When present, *Elsinoë* spp. account for extensive disease symptoms, probably in partnership with other fungi. Therefore, the fungal microbiome associated with cassava superelongation disease should be further examined to identify the fungi it includes, their interactions, their products, and their role in causing the disease.

## Figures and Tables

**Table 1:** Symptomatic cassava leaf samples used for fungal and DNA extraction from BB, JA, SVG, and TT.

Country	Field Name	Sample ID	Local Plant Name	Location Latitude/Longitude
BB	Haymans	BBH1	Red stick	13.241298/−59.6288592
	Dukes	BBD2	Red stick	13.1901331/59.5916546
	CARDI	BBC3	Red stem	13.076014/−59.5712249
	Valley	BBV4	Red stick	13.1290087/59.5663905
	Three Houses	BBT5	Red stick	13.1581469/59.4597395
	Searles	BBS6	Red stick	13.0901029/59.5173527
	Fisherpond	BBF7	White Stick	13.16639/−59.554403
JA	Wallen	JAWa1	CM849	17.9450046/76.9167181
	Wallen	JAWa2	MCOL-1505	17.9613046/76.9213466
	Windsor	JAWi3	Bra 383	18.3590873/77.6506805
	Bernard Lodge	JABL4	Bra 383	17.9740417/76.9264224
	St Elizabeth	JAE5	White Stem	18.0515321/−77.759791
	St Elizabeth	JAE6	White Stem	18.0515321/−77.759791
	Montpelier	JAMP7	MCOL-1505	18.3629545/77.9239346
TT	Freeport Centeno	TTF3	CIAT	10.4512865/61.4052487
		TTCe4	Butter stick	10.5980403/−61.318302
	Penal	TTPe5	MMEX	10.156948/−61.4336586
SVG	Queens Drive	SVGQ2	Bowes	13.1615637/61.2035787
	Layou	SVGL3	^a^ NA	13.2005488/61.2625552
	Bequia	SVGB4	Bitter (white stem)	13.0164531/61.2453087
	Troumaca	SVGT5	Butter stick	13.2612914/61.2431145

^a^ NA—Variety name not available.

**Table 2:** Disease severity scale of superelongation disease in cassava.

Scale	Symptoms
0.0	No plants with visible symptoms.
1.0	Minute flecks on the leaf surface.
1.5	Defined, circular chlorotic spots on the leaf surface.
2.0	Cankers on leaves or petioles.
3.0	Cankers on leaves, petioles, and stems, severe leaf distortion.
4.0	Elongation, cankers on leaves, petioles and stems, severe leaf distortion and scorching.

**Table 3:** Shannon diversity index of the cassava leaf mycobiome in four Caribbean islands.

Island	Sample	N	Shannon
BB	BBH1	10	1.73
	BBD2	8	1.49
	BBC3	6	1.14
	BBV4	9	1.77
	BBT5	7	1.06
	BBS6	11	1.55
	BBF7	4	0.96
SVG	SVGQ2	10	1.77
	SVGL3	17	2.38
	SVGB4	8	1.84
	SVGT5	4	0.69
TT	TTF3	13	1.71
	TTCe4	12	1.96
	TTPe5	8	0.77
JA	JAWa1	6	1.04
	JAWa2	6	0.70
	JAWi3	10	1.69
	JABL4	9	1.80
	JAE5	15	2.56
	JAE6	15	2.34
	JAMP7	6	0.65

**Table 4:** Geolocational differences of two fungal genera in the cassava mycobiome in the Caribbean islands.

Fungi	Location	Base Mean	log2 Fold Change	Stat	*p*-Value
*Neofusicoccum*	BB-JA	10.76	25.06	6.93	4.30 × 10^−12^
*Neofusicoccum*	BB-SVG	10.76	22.17	5.19	2.05 × 10^−7^
*Neofusicoccum*	BB-TT	10.76	20.83	4.42	9.84 × 10^−6^
*Alternaria*	JA-SVG	56.38	23.70	9.09	9.94 × 10^−20^
*Alternaria*	TT-SVG	56.38	19.19	6.15	7.60 × 10^−10^
*Alternaria*	BB-SVG	56.38	22.59	8.66	4.56 × 10^−18^

## Data Availability

The data for this study were deposited in the European Nucleotide Archive (ENA) at EMBL-EBI under accession number PRJEB46406 (https://www.ebi.ac.uk/ena/browser/view/PRJEB46406, accessed on 5 January 2022).

## Supplementary Materials

The following supporting information can be downloaded at: https://www.mdpi.com/article/10.3390/jof9121130/s1, Figure S1: Relative abundance of fungal phyla from symptomatic cassava leaf samples from BB, JA, SVG, and TT. Figure S2: Heatmap showing the class composition by OTUs in infected cassava from BB, JA, SVG, and TT.
