# Supplementary material for: Characterization of the Cassava Mycobiome in Symptomatic Leaf Tissues Displaying Cassava Superelongation Disease
Source: J Fungi (Basel). 2023 Nov 23;9(12):1130. doi: 10.3390/jof9121130 (PMC10743849; doi:10.3390/jof9121130)
Supplement: Supplementary file 1 [file jof-09-01130-s001.zip › jof-2689894-supplementary.pdf]

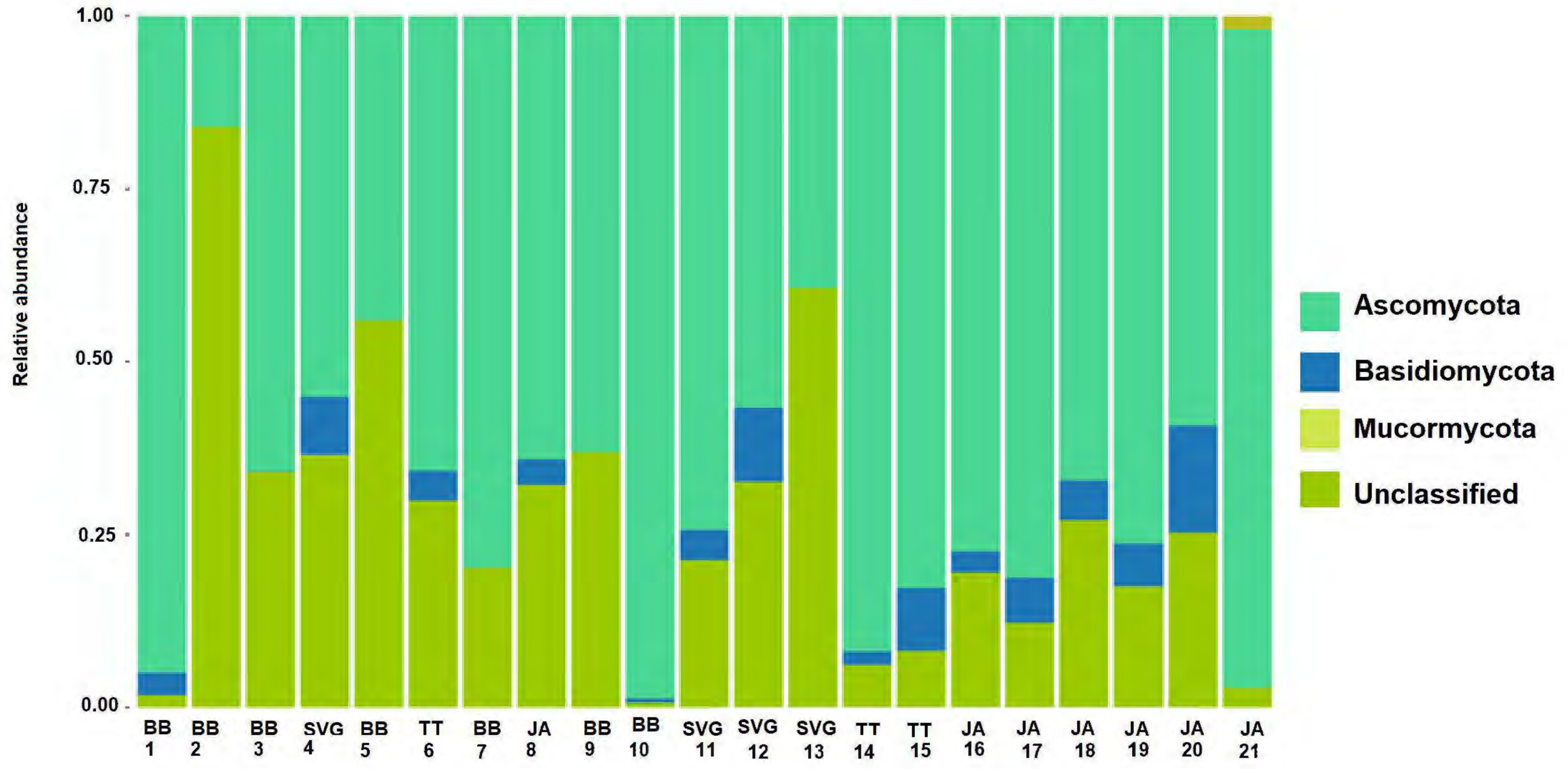

Supplemental Figure S1- Relative abundance of fungal phyla from symptomatic cassava leaf samples from BB, JA, SVG and TT.

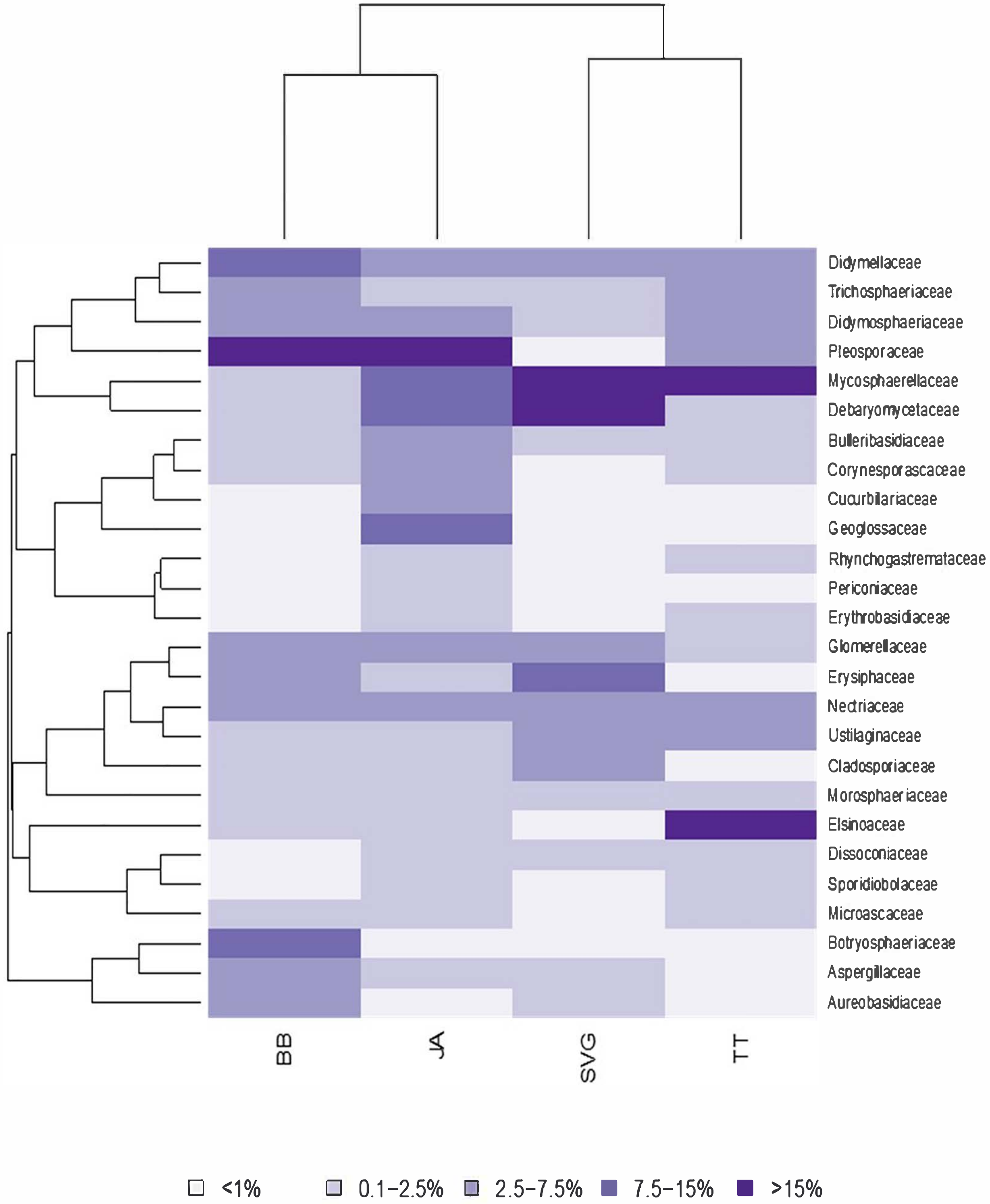

Supplemental Figure S2: Heatmap showing the class composition by OTUs in infected cassava from BB, JA, SVG and TT.
